# Potato tuber expression of Arabidopsis *WRINKLED1* increase triacylglycerol and membrane lipids while affecting central carbohydrate metabolism

**DOI:** 10.1111/pbi.12550

**Published:** 2016-03-17

**Authors:** Per Hofvander, Till Ischebeck, Helle Turesson, Sandeep K. Kushwaha, Ivo Feussner, Anders S. Carlsson, Mariette Andersson

**Affiliations:** ^1^ Department of Plant Breeding Swedish University of Agricultural Sciences Alnarp Sweden; ^2^ Department of Plant Biochemistry Albrecht‐von‐Haller Institute Georg‐August‐University Goettingen Germany; ^3^ PlantLink Department of Plant Protection Biology Swedish University of Agricultural Sciences Alnarp Sweden

**Keywords:** *Solanum tuberosum*, tuber, WRINKLED1, lipid, triacylglycerol, starch, metabolite

## Abstract

Tuber and root crops virtually exclusively accumulate storage products in the form of carbohydrates. An exception is yellow nutsedge (*Cyperus esculentus*) in which tubers have the capacity to store starch and triacylglycerols (TAG) in roughly equal amounts. This suggests that a tuber crop can efficiently handle accumulation of energy dense oil. From a nutritional as well as economic aspect, it would be of interest to utilize the high yield capacity of tuber or root crops for oil accumulation similar to yellow nutsedge. The transcription factor WRINKLED1 from *Arabidopsis thaliana*, which in seed embryos induce fatty acid synthesis, has been shown to be a major factor for oil accumulation. WRINKLED1 was expressed in potato (*Solanum tuberosum*) tubers to explore whether this factor could impact tuber metabolism. This study shows that a WRINKLED1 transcription factor could induce triacylglycerol accumulation in tubers of transformed potato plants grown in field (up to 12 nmol TAG/mg dry weight, 1% of dry weight) together with a large increase in polar membrane lipids. The changes in metabolism further affected starch accumulation and composition concomitant with massive increases in sugar content.

## Introduction

Plants synthesize and deposit storage products as energy to be utilized after a shorter or longer time frame. Long‐term storage is primarily in organs responsible for new generations as seeds or tubers and roots. These storage compounds vary among species but are usually present in the form of starch, polyfructose, sucrose, oil and protein. Oil is the most energy dense form of storage and mainly found in seeds and the mesocarp of some fruits. Plant species with underground stem tubers and roots generally store carbohydrates in different forms in such sink organs. An exception to this is yellow nutsedge (*Cyperus esculentus*) that besides carbohydrates, store triacylglycerols (TAG) up to around 15% of fresh weight (Linssen *et al*., 1989). Additionally, it stores almost 20% starch of fresh weight in its tuber parenchyma tissue which, on a combined quantitative basis, is very uncommon among plants. This unique example shows that underground storage organs have the capacity to accumulate significant amounts of TAG.

A transcription factor which expression has been found to be closely connected to oil accumulation is *WRINKLED1* from *Arabidopsis thaliana* (Focks and Benning, 1998). It belongs to the AP2/EREB domain family and triggers the expression of genes involved in glycolysis and fatty acid synthesis (Cernac and Benning, 2004; Focks and Benning, 1998). Expression of maize homologs complementing the WRINKLED1 mutation in Arabidopsis and heterologous expression in leaf tissue of Arabidopsis have shown WRINKLED1 to have the capacity for complementation of deficiencies across species as well as to ectopically induce oil accumulation and heterologously enhance oil content in seeds (An and Suh, 2015; Pouvreau *et al*., 2011; Sanjaya *et al*., 2011; Yang *et al*., 2015). Recently, also protein structural properties have been investigated to elucidate and enhance the function of the WRINKLED1 transcription factor (Ma *et al*., 2015). Of several known transcription factors of importance for oil accumulation in seed embryos, only a *WRINKLED1* homolog was highly up‐regulated in mesocarp of oil palm (*Elaeis guineensis*) compared to date palm (*Phoenix dactylifera*) (Bourgis *et al*., 2011). This could indicate a different regulating mechanism dominated by WRINKLED1 in other oil accumulating tissues than seed embryo.

Tubers are sink organs with a massive sucrose import, which is similar to the situation in developing seeds and fruits. As oil accumulation is such a rare event in tubers, but evidently can coexist with starch deposition, we were interested to assess whether tubers that only store starch can accommodate TAG accumulation and whether the WRINKLED1 transcription factor can cause such a metabolic change in an underground storage tissue as stem tubers of potato.

It is not only of scientific interest to investigate how a crop not evolved for a dual storage accumulation accommodates additional up‐regulated pathways but could also be of importance from a crop development perspective. Root and tuber crops generally have very high yield although a large part of the harvest is water and only 25–30% is dry matter (www.fao.org/es/faodef/fdef02e.htm). This could be compared with yellow nutsedge that has up to 60% dry matter (Turesson *et al*., 2010). Adding TAG deposition to typical carbohydrate crops would increase the nutritional density of staple crops that currently provide an exclusive carbohydrate diet. Potato is not only an important food crop but also the main crop used for starch production in northern Europe (www.starch.eu). If TAG is accumulated to substantial amounts in potatoes, it would also be valuable to recover the oil portion besides processing the starch.

In this study, we found *WRINKLED1* expression to dramatically change metabolism and induce TAG accumulation in tubers. Polar and membrane lipids were found to be greatly increased in transgenic potato tubers concomitant with TAG accumulation.

## Results

### Gene construct and transformation for *AtWRI1* expression in potato tubers

A synthetically produced gene corresponding to the *WRINKLED1* coding region of *Arabidopsis thaliana* (At3 g54320) (*AtWRI1*) was cloned after a *GBSS* promoter (*Solanum tuberosum*) which has been characterized as tuber specific (Visser *et al*., 1991). Transformation was performed on potato variety Kuras, and putative transgenic shoots were selected on regeneration medium containing kanamycin. Based on PCR analysis, 45% of analysed shoots showed T‐DNA integration (results not shown) and individual shoots were selected for propagation of *in vitro* microtubers. Lipids were extracted from microtubers of ten individual transgenic lines and analysed for changes in content of neutral lipids in comparison with Kuras *in vitro* microtubers. Several lines displayed an increase in triacylglycerol (TAG) content, of which lines 8001, 8003, 8016 and 8022 were selected for more detailed studies (Figure S1). TAG content of Kuras microtubers was found to be 0.1 mg/g dw while a content of more than 20 mg/g dw (2% TAG of dry weight) could be measured for individual transgenic microtubers (Figure S2). Consistent for TAG from selected transgenic microtubers was a change in fatty acid (FA) composition with an increase in 18:2 and 18:1 at the expense of 18:3 (Figure S3). All the selected transgenic lines contained multiple inserts of the T‐DNA (Figure S4).

### AtWRI1 induces oil accumulation in soil grown tubers

**Figure 1:**
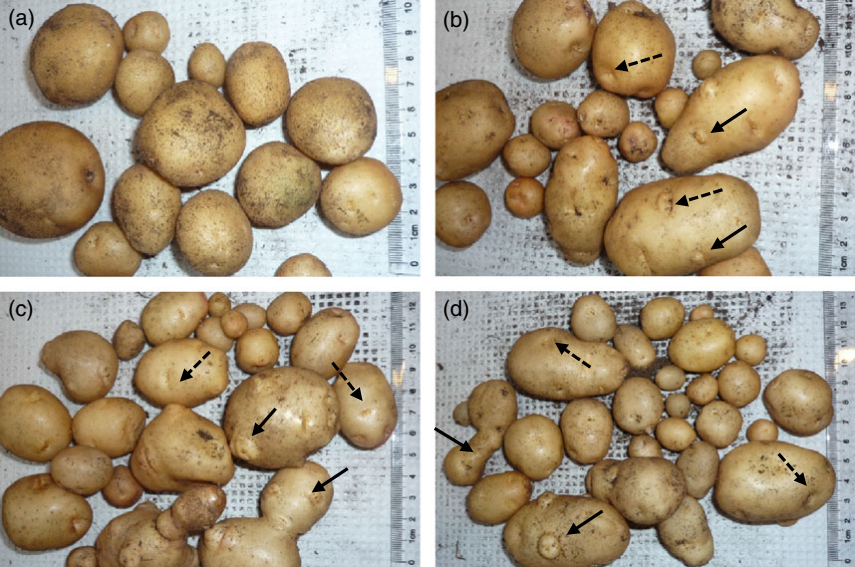
Phenotype of greenhouse grown tubers, (a) Kuras, (b) 8001, (c) 8003, (d) 8016. Solid arrows indicate secondary tuber developments and dashed arrows indicate deeper eyes of transgenic tubers.

**Figure 2:**
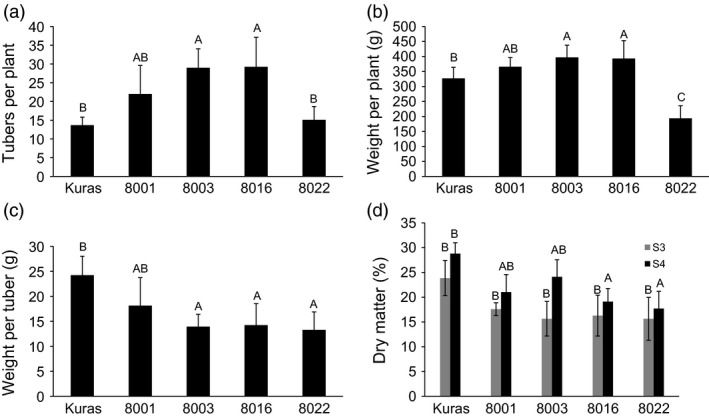
Greenhouse tubers from Kuras and transformed lines after stage S4 (a‐c) and stage S3 compared to S4 (d). (a) Number of tubers per plant, (b) Tuber mass per plant, (c) Mass per tuber, (d) Dry matter content at stage S3 and S4. Letters distinguish significant different means according to Tukey's test at level *P* < 0.05.

Cuttings of Kuras and lines 8001, 8003, 8016 and 8022 were transferred to soil in the greenhouse. Growth and development of transgenic lines were indistinguishable from control with the exception for line 8022 which appeared erect, thin in growth and with small curly leaves. Greenhouse cultivation lasted for 4 months, and sampling was performed after 2 (S2), 3 (S3) and 4 months (S4). After 4 months, the haulm was cut and watering stopped 2 weeks prior to harvesting. All transgenic lines displayed altered tuber morphology. The parental variety Kuras has rounded and slightly flattened tuber morphology, while tubers of the transgenic lines generally were slightly elongated, had deeper eyes and were rich in secondary tuber developments (Figure 1). Transgenic lines, with the exception of line 8022, on average produced more tubers than wild type but of less average weight (Figure 2). Total tuber mass production per plant showed a slight average increase with the exception for line 8022 (Figure 2). Line 8022 was excluded from further studies and generalized conclusions as it displayed aberrant growth. Dry matter content was on average less for transgenic lines compared to control but increased for both control and transgenic lines between S3 and S4 (Figure 2).

**Figure 3:**
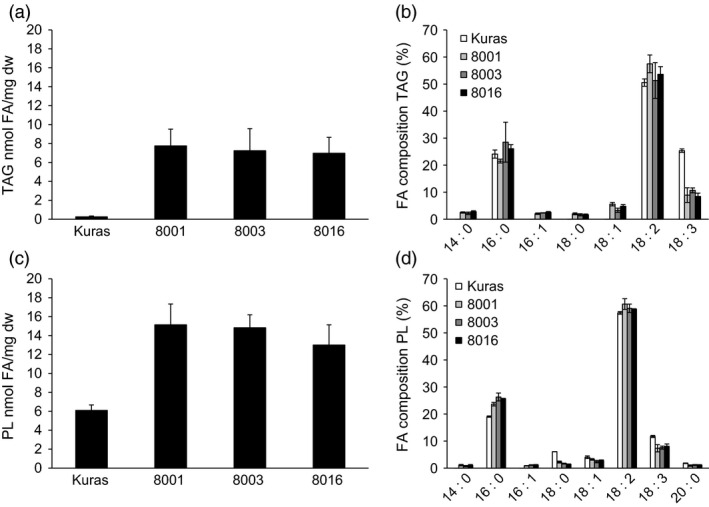
Analysis of triacylglycerols (TAG) and polar lipids (PL) of greenhouse grown tubers, (a) TAG content of tubers of Kuras and transgenic lines, (b) Fatty acid composition of TAG from Kuras and transgenic lines, (c) PL content of tubers of Kuras and transgenic lines, (d) Fatty acid composition of PL from Kuras and transgenic lines. Three biological replicates ±SD.

TAG had increased up to 20‐fold, 3 mg/g dw over control tubers at the time of sampling after 4 months (Figure 3a). However, an intermediate sampling (S3), made after 3 months to represent a metabolic status of active development, showed that the TAG content decreased between S3 and S4 (results not shown). TAG composition analysis revealed a large decrease in 18:3 levels among FA contained in TAG (Figure 3b). Fatty acids associated with polar lipids increased in transgenic lines with a similar absolute amount as for TAG but with no obvious alterations in fatty acid composition (Figure 3c,d).

### Expression of *AtWRI1* directly or indirectly affects carbohydrate metabolism including starch

Metabolites and starch were analysed for the S3 stage, which represents tubers in an active accumulation phase. Transgenic lines showed a 10–18% decreased starch content of dry weight compared to the parental variety at this stage (Table 1).

**Table 1:** Starch and metabolite concentrations of greenhouse tubers from Kuras control and transgenic lines 8001, 8003 and 8016 sampled at stage S3, representing sampling 3 months postplanting

mg/g DW	Kuras	8001	8003	8016
Starch	801.7 ± 26.0	681.1 ± 44.3	722.4 ± 42.1	658.7 ± 31.5
Sucrose	6.3 ± 1.0	19.9 ± 3.4	15.2 ± 7.0	19.4 ± 4.0
Glucose	2.2 ± 1.3	98.4 ± 18.3	107.2 ± 72.9	184.0 ± 25.3
Fructose	0.14 ± 0.11	1.33 ± 1.13	3.46 ± 2.22	5.15 ± 1.56
Glucose‐6‐P	0.10 ± 0.02	0.17 ± 0.07	0.17 ± 0.04	0.20 ± 0.04
Fructose‐6‐P	0.030 ± 0.007	0.041 ± 0.016	0.039 ± 0.008	0.046 ± 0.008
Pyruvate	0.014 ± 0.003	0.017 ± 0.003	0.017 ± 0.004	0.018 ± 0.003
Citrate	11.68 ± 1.30	5.38 ± 0.11	4.35 ± 1.65	2.84 ± 0.52
Isocitrate	0.18 ± 0.02	0.05 ± 0.02	0.05 ± 0.02	0.04 ± 0.00
Ketoglutarate	0.011 ± 0.004	0.012 ± 0.003	0.020 ± 0.004	0.016 ± 0.005
Succinate	0.010 ± 0.008	0.014 ± 0.003	0.027 ± 0.023	0.024 ± 0.004
Fumarate	0.010 ± 0.003	0.005 ± 0.001	0.009 ± 0.002	0.007 ± 0.000
Malate	3.66 ± 0.84	7.56 ± 3.97	14.08 ± 1.13	12.14 ± 1.26
Glycerol‐3‐P	0.006 ± 0.001	0.017 ± 0.003	0.033 ± 0.013	0.038 ± 0.010
Glycerol	0.18 ± 0.03	0.20 ± 0.01	0.21 ± 0.01	0.19 ± 0.01

Metabolites were extracted and investigated for differential concentration in transgenic lines compared to parental genotype. Sugars as sucrose, glucose and fructose were greatly increased in transgenic tubers (Table 1). Most prominent was the increase in glucose which amounted to up to 100‐fold higher (18.4% of dw) than in the parental genotype. Fructose was increased up to 37‐fold (0.5% of dw). TCA cycle intermediates were also affected with a fourfold increase of malic acid or decrease of citric and isocitric acid, respectively (Table 1). Amino acids as alanine and valine were found increased while asparagine was decreased (Dataset S1). The backbone for TAG assembly, glycerol‐3‐P, was also increased in the transgenic lines (Table 1).

### Transcriptome analysis reveals differential gene expression of transgenic tubers

Of interest to investigate were pathways leading from sucrose to precursors for fatty acid synthesis and subsequent fatty acid modification and assembly into complex lipids. Line 8016 was selected for comparative analysis at three different time points with the parental genotype. Sequenced reads corresponding to *AtWRI1* could be detected at all three stages of development in line 8016 while very low levels of *AtWRI1* were detected in the parental control (results not shown). Several genes involved in late steps of plastid glycolysis were found to be up‐regulated in transgenic tubers (Table 2). Genes for plastid localized glycolysis encoding phosphoglyceromutase, enolase and pyruvate kinase were up‐regulated (12‐, 7‐ and 6‐fold, respectively, at S3). Also transcripts encoding plastidic NADP‐malic enzyme were up‐regulated (10‐fold at S3), which could provide an alternate route to supplying pyruvate. In line with these transcripts, encoding plastid dicarboxylate transporters, potentially of importance for malate transport, were up‐regulated. All transcripts encoding subunits of plastidic pyruvate dehydrogenase were up‐regulated (up to 27‐fold at S3) indicating a push towards acetyl‐CoA production. Cytosolic ATP‐citrate lyase transcripts were also found up‐regulated (up to 22‐fold at S3) (Table 3) which could provide additional supply in the direction of pyruvate via oxaloacetate and malate but also acetyl‐CoA as substrate for the mevalonate pathway. A transcript annotated as a phosphoenolpyruvate (PEP) transporter (PPT) was up‐regulated (sevenfold at S3) indicating an enforcement of plastid PEP import and subsequent plastid enzymatic reactions leading to an increased pyruvate supply for fatty acid synthesis (Table 2). The major potato tuber plastid transporter for carbohydrate import, glucose‐6‐phosphate/phosphate translocator (GPT), showed essentially unchanged transcriptional expression level while another contig representing a minor form was up‐regulated although to a low level of expression. A gene encoding a transporter (GlcT) defined as exporting glucose was up‐regulated at later stages of development.

**Table 2:** Transcripts found differentially expressed and annotated as belonging to given specific pathways in line 8016 compared to Kuras control. Contig names are given with annotated function and closest Arabidopsis homolog. Expression is given as fragments per kilobase of exon per million fragments mapped (FPKM). Differential expression is given as fold difference using the quote between 8016 and Kuras FPKM with significant differences highlighted in bold. S2, S3 and S4 represent samplings 2‐, 3‐ and 4‐month postplanting

Contig		Location	Kuras (FPKM)			8016 (FPKM)			8016/Kuras			At locus
	Function		S2	S3	S4	S2	S3	S4	S2	S3	S4	
	Plastid transporter											
c22973_g1_i1	Glucose‐6‐phosphate/phosphate translocator	Plastid	492.69	740.72	365.80	477.90	874.80	870.01	0.97	1.18	2.38	AT1G61800
c28539_g1_i1	Glucose‐6‐phosphate/phosphate translocator	Plastid	0.04	0.23	0.09	7.54	19.67	15.50	**196.69**	**84.77**	**181.64**	AT1G61800
c22725_g1_i1	Glucose transporter	Plastid	36.32	18.40	10.85	47.97	85.58	50.57	1.32	**4.65**	**4.66**	AT5G16150
c28098_g1_i1	Phosphoenolpyruvate transporter	Plastid	23.74	14.67	9.84	65.31	105.47	70.35	2.75	**7.19**	**7.15**	AT5G33320
c24279_g1_i1	Triosephosphate transporter	Plastid	0.01	0.07	0.14	4.91	11.49	4.16	**737.10**	**159.58**	**29.51**	AT5G46110
c30397_g1_i1	Dicarboxylate transporter 1	Plastid	16.92	8.45	8.24	30.10	40.66	28.17	1.78	**4.81**	3.42	AT5G12860
c27866_g1_i1	Dicarboxylate transporter 2	Plastid	7.49	4.99	3.69	23.31	44.29	23.96	3.11	**8.87**	**6.50**	AT5G64290
	Glycolysis											
c23236_g1_i1	Pyruvate kinase	Plastid	173.22	225.37	263.82	1850.35	1491.42	1735.27	**10.68**	**6.62**	**6.58**	AT3G22960
c25285_g1_i1	Pyruvate kinase	Plastid	130.08	122.33	114.21	619.03	672.59	651.27	**4.76**	**5.50**	**5.70**	AT5G52920
c29576_g1_i5	Phosphoglycerate kinase	Plastid	1.45	1.54	1.37	7.65	7.92	5.60	**5.27**	**5.16**	4.09	AT5G61450
c29147_g1_i1	Phosphoglyceromutase	Plastid	6.74	2.55	2.01	28.10	31.90	23.49	4.17	**12.50**	**11.69**	AT1G22170
c25121_g1_i1	Enolase	Plastid	75.68	58.54	35.77	317.76	423.82	329.56	4.20	**7.24**	**9.21**	AT1G74030
	Hexose‐P											
c26088_g3_i1	Phosphoglucoisomerase	Plastid	21.35	10.21	6.19	29.70	47.10	31.84	1.39	**4.61**	**5.15**	AT4G24620
c64003_g1_i1	Hexokinase	Plastid	16.31	13.42	11.01	41.59	64.06	42.76	2.55	**4.77**	3.88	AT1G47840
c28483_g1_i1	Fructokinase	Plastid	10.06	4.74	3.13	22.58	33.30	25.08	2.25	**7.03**	**8.02**	AT1G66430
	OPPP											
c26268_g1_i1	Transketolase	Plastid	104.32	72.76	38.55	186.85	297.64	216.86	1.79	4.09	**5.63**	AT2G45290
c28692_g1_i1	Transaldolase	Plastid	40.45	31.48	18.26	140.33	224.41	144.12	3.47	**7.13**	**7.89**	AT1G12230
	Malate, pyruvate metabolism											
c16822_g1_i1	Malate dehydrogenese	Cytosol	0.88	1.01	0.84	6.21	4.23	4.07	**7.06**	**4.19**	**4.85**	AT1G04410
c11587_g1_i1	Malate dehydrogenese	Mitochondria	9.50	8.64	6.17	19.21	33.85	28.82	2.02	3.92	**4.67**	AT1G53240
c24212_g1_i1	Malate dehydrogenese	Plastid	1.33	1.37	2.29	21.01	35.88	27.97	**15.81**	**26.21**	**12.21**	AT3G47520
c11332_g1_i1	Malic enzyme (NADP)	Plastid	17.99	13.97	23.57	123.84	146.77	112.30	**6.88**	**10.51**	**4.76**	AT1G79750
	Calvin cycle											
c21104_g1_i1	Rubisco small subunit	Plastid	0.04	0.09	0.06	264.93	354.40	318.00	**6161.05**	**3967.19**	**5579.03**	AT1G67090
	Sucrose metabolism											
c19348_g1_i1	Invertase	Cytosol	0.15	0.10	0.11	4.22	9.48	5.24	**27.70**	**90.82**	**48.11**	AT4G34860
	Starch metabolism											
c30369_g1_i1	Starch synthase IV	Plastid	1.63	1.00	0.86	7.17	7.26	5.14	4.41	**7.27**	**5.96**	AT4G18240

**Table 3:** Transcripts found differentially expressed and annotated as belonging to given specific pathways in line 8016 compared to Kuras control. Contig names are given with annotated function and closest *Arabidopsis thaliana* homolog. Expression is given as fragments per kilobase of exon per million fragments mapped (FPKM). Differential expression is given as fold difference using the quote between 8016 and Kuras FPKM with significant differences highlighted in bold. S2, S3 and S4 represent samplings 2‐, 3‐ and 4‐month postplanting

Contig		Location	Kuras (FPKM)			8016 (FPKM)			8016/Kuras			At locus
	Function		S2	S3	S4	S2	S3	S4	S2	S3	S4	
	Fatty acid synthesis											
c19523_g1_i1	Pyruvate Dehydrogenase, E1a component	Plastid	30.81	14.97	15.69	195.58	359.01	222.29	**6.35**	**23.99**	**14.17**	AT1G01090
c29557_g1_i3	Pyruvate Dehydrogenase, E1b component	Plastid	8.70	6.08	5.80	44.55	76.19	51.43	**5.12**	**12.53**	**8.86**	AT2G34590
c85823_g1_i1	Pyruvate Dehydrogenase, E1b component	Plastid	5.11	2.42	2.84	21.63	38.91	26.93	4.23	**16.08**	**9.50**	AT1G30120
c28567_g1_i1	Dihydrolipoamide Acetyltransferase, E2 component	Plastid	16.63	9.37	6.81	65.01	127.14	70.57	3.91	**13.57**	**10.37**	AT1G34430
c22404_g1_i1	Dihydrolipoamide Acetyltransferase, E2 component	Plastid	22.12	7.72	4.61	111.44	210.94	144.01	**5.04**	**27.33**	**31.24**	AT3G25860
c24969_g1_i1	Dihydrolipoamide Dehydrogenase, E3 component	Plastid	50.09	27.86	12.19	227.91	323.14	235.45	4.55	**11.60**	**19.32**	AT3G16950
c85642_g1_i1	Carboxyltransferase; Subunit of Heteromeric ACCase	Plastid	46.93	34.60	19.94	147.42	235.44	166.90	3.14	**6.80**	**8.37**	AT2G38040
c21469_g1_i1	Biotin Carboxyl Carrier Protein of Heteromeric ACCase	Plastid	19.10	9.69	7.96	83.83	156.73	108.14	**4.39**	**16.18**	**13.59**	AT5G15530
c27158_g1_i1	Biotin Carboxyl Carrier Protein of Heteromeric ACCase	Plastid	7.37	3.47	2.50	11.14	14.84	12.12	1.51	**4.28**	**4.85**	AT5G16390
c20279_g1_i1	Biotin Carboxyl Carrier Protein‐like	Plastid	18.69	20.34	16.65	105.82	129.13	148.45	**5.66**	**6.35**	**8.92**	AT3G15690
c19931_g1_i1	Biotin Carboxylase of Heteromeric ACCase	Plastid	51.37	23.13	14.52	213.19	384.39	247.91	**4.15**	**16.62**	**17.08**	AT5G35360
c23829_g1_i1	Malonyl‐CoA: ACP Malonyltransferase	Plastid	39.62	24.22	13.63	187.07	244.07	215.71	**4.72**	**10.08**	**15.83**	AT2G30200
c24708_g2_i1	Acyl Carrier Protein 3	Plastid	94.80	58.05	42.92	311.47	449.23	387.22	3.29	**7.74**	**9.02**	AT1G54630
c24708_g1_i1	Acyl Carrier Protein 5	Plastid	124.18	69.96	46.84	463.41	972.60	616.92	3.73	**13.90**	**13.17**	AT5G27200
c23512_g1_i1	Ketoacyl‐ACP Synthase I	Plastid	32.15	13.01	9.43	146.19	209.88	160.92	**4.55**	**16.13**	**17.07**	AT5G46290
c24075_g1_i1	Ketoacyl‐ACP Synthase III	Plastid	26.17	14.12	9.32	47.49	90.05	54.64	1.82	**6.38**	**5.86**	AT1G62640
c16521_g1_i1	Enoyl‐ACP Reductase	Plastid	22.25	10.97	6.42	131.63	248.04	177.77	**5.92**	**22.60**	**27.69**	AT2G05990
c27994_g1_i1	Ketoacyl‐ACP Reductase	Plastid	6.60	5.45	4.38	135.57	140.79	149.50	**20.55**	**25.86**	**34.15**	AT1G24360
c8651_g1_i1	Hydroxyacyl‐ACP Dehydrase	Plastid	47.72	26.76	12.55	135.26	250.54	178.58	2.83	**9.36**	**14.23**	AT5G10160
c42396_g1_i1	Stearoyl‐ACP Desaturase	Plastid	14.72	16.09	19.85	178.63	234.00	274.00	**12.14**	**14.54**	**13.80**	AT3G02630
c18530_g1_i1	Stearoyl‐ACP Desaturase	Plastid	26.85	13.79	5.86	49.18	112.79	69.10	1.83	**8.18**	**11.78**	AT2G43710
c30524_g3_i1	ATP‐citrate lyase A‐1	Cytosol	6.47	2.67	1.33	7.19	24.29	9.58	1.11	**9.10**	**7.23**	AT1G10670
c11428_g1_i1	ATP‐citrate lyase A‐2	Cytosol	18.76	7.46	5.02	22.43	74.59	21.90	1.20	**9.99**	**4.36**	AT1G60810
c11638_g1_i1	ATP‐citrate lyase B‐1	Cytosol	5.34	2.13	1.47	33.98	46.80	33.96	**6.37**	**22.02**	**23.14**	AT3G06650
c74426_g1_i1	ATP‐citrate lyase B‐2	Cytosol	3.52	2.33	1.58	31.26	44.49	32.80	**8.87**	**19.13**	**20.77**	AT5G49460
	TAG synthesis											
c23789_g1_i1	NAD‐dependent Glycerol‐3‐Phosphate Dehydrogenase	Cytosol	3.53	2.81	6.45	20.68	24.79	16.48	**5.86**	**8.83**	2.56	AT2G41540
	Other lipid related functions											
c20364_g1_i1	Phosphatidylcholine:diacylglycerol cholinephosphotransferase		18.11	14.19	13.62	56.43	81.03	40.26	3.12	**5.71**	2.96	AT3G15820
c5504_g1_i1	Phosphoethanolamine N‐Methyltransferase	Cytosol	1.01	0.24	3.38	19.59	39.49	15.58	**19.43**	**165.69**	**4.60**	AT3G18000
c14168_g1_i1	Acyl‐CoA Binding Protein 6	Cytosol	119.90	108.64	176.64	842.84	757.29	653.71	**7.03**	**6.97**	3.70	AT1G31812
c26561_g1_i1	PII protein	Plastid	19.55	10.34	7.59	131.52	238.76	148.83	**6.73**	**23.10**	**19.62**	AT4G01900
c27412_g1_i1	Long‐Chain Acyl‐CoA Synthetase 9	Plastid?	15.72	12.07	7.79	68.62	94.83	72.80	**4.36**	**7.86**	**9.35**	AT1G77590
c22461_g1_i1	Oleate Desaturase	ER	151.53	258.47	79.59	1560.43	2228.04	1960.82	**10.30**	**8.62**	**24.64**	AT3G12120
c19969_g1_i1	Oleosin 3	Oilbody	0.53	1.51	0.45	9.97	32.44	9.24	**18.96**	**21.48**	**20.77**	AT5G51210
c1519_g1_i1	Oleosin 1	Oilbody	0.42	0.88	0.40	8.38	30.87	8.03	**19.99**	**35.22**	**19.90**	AT4G25140
c9835_g1_i1	Oleosin 2	Oilbody	0.53	1.39	2.00	8.06	8.99	4.80	**15.21**	**6.45**	2.40	AT5G40420
c19906_g1_i1	LEC1	Nucleus	0.03	0.01	0.17	3.72	18.88	7.51	**112.70**	**1953.21**	**43.52**	AT1G21970

The most highly up‐regulated transcript among genes related to metabolic cycles for substrate supply and processing was annotated as encoding a small subunit of ribulose‐1,5‐bisphosphate carboxylase/oxygenase (Rubisco), which was found having an 4000‐fold increase at S3 (Table 2). Several genes encoding products where the closest homologs in Arabidopsis are annotated as belonging to the pentose phosphate pathway as transaldolase and transketolase were also found to be up‐regulated (7‐ and 4‐fold, respectively, at S3), which could indicate an increased expression of genes for supplying additional substrates for pyruvate synthesis via hexose phosphates to ribulose‐1,5‐bisphosphate and further metabolization through late parts of glycolysis.

All essential transcripts for plastidic fatty acid synthesis were found to be up‐regulated (Table 3). Interestingly, also genes involved in fatty acid modifications and processing were found up‐regulated like transcripts annotated as stearoyl‐ACP desaturase, acyl‐ACP thioesterase and oleate desaturase. Only few genes involved in complex lipid assembly and control were found to be up‐regulated, with examples of genes as phosphatidylcholine:diacylglycerol cholinephosphotransferase (PDCT) and transcripts annotated as oleosins (fivefold and up to 35‐fold, respectively, at S3).

A transcript with homology to LEC1 was found up‐regulated (more than 1000‐fold at S3), which could be connected to the up‐regulation of transcripts downstream of fatty acid synthesis (Table 3).

No transcripts related to β‐oxidation or starch degradation were found to be up‐regulated except for a contig annotated as cytosolic invertase although the final expression levels were still in a low range in comparison with other genes.

### Structural studies show presence of oil droplets and changes of starch granules

**Figure 4:**
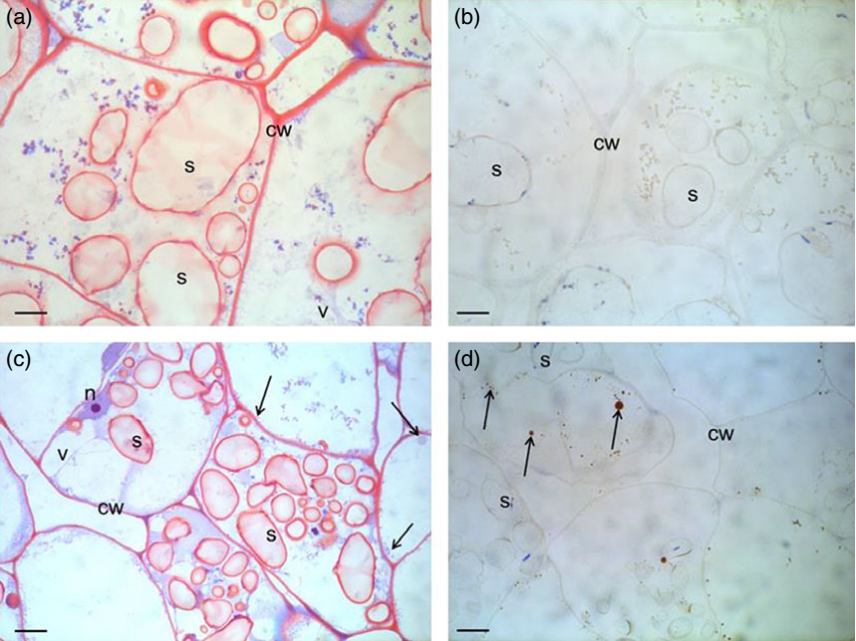
Light micrographs of Kuras (a,b) and transgenic potato line 8016 (c,d). (a,c) Overview staining with MAS (Triple staining Methylene blue—Azur A—Safranin O) and (b, d) Oil specific staining, Sudan black. Arrows indicate oil droplets, s—starch granule, v—vacuole, cw—cell wall, n—nuclei. Scale bars 10 μm.

**Figure 5:**
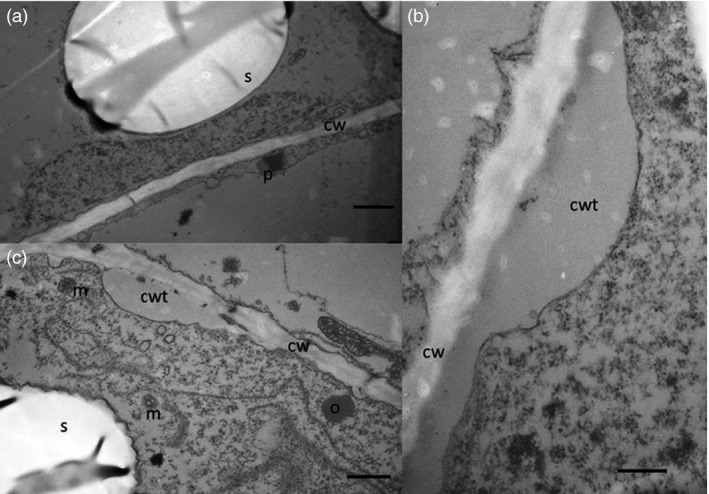
Transmission electron micrographs of potato tubers. (a) Kuras, scale bar 1 μm, (b) 8016 scale bar 1 μm, (c) 8016 scale bar 500 nm. S—starch granule, cw—cell wall, cwt—cell wall thickness, o—oil droplet, m—mitochondria, p—peroxisome.

The cell structure of the tubers was studied under light microscope and transmission electron microscope (TEM) to examine and compare storage cells of the wild‐type and transgenic tubers. Transgenic material displayed smaller and irregularly shaped starch granules compared to the wild type (Figure 4a,c). Oil droplets were detected along the cell walls, which could not be found in the wild type (Figure 4b,d). Furthermore, impact on membranes due to increased amounts of membrane lipids was noted. TEM micrographs revealed clear membrane invaginations (abbreviated as CWT) in the transgenic line (Figure 5).

### Expression of *AtWRI1* in field grown tubers affects tuber yield, size and phenotype

In a field trial, Kuras, 8001, 8003, 8016 and 8022, were grown in four rows of 13–15 plants per row. Lines 8001, 8003 and 8016 could not be distinguished from the parental variety. No differences in emergence, plant morphology, haulm height and size, maturation, wilting or susceptibility to diseases could be observed. The tuber fresh weight yield at maturity was found to be higher for two of the transgenic lines, 8001 and 8016 with 13 and 27 percentage increase, respectively, compared to the parental variety, while line 8003 had 28 percentage points lower yield (Table 4).

**Table 4:** Total field trial tuber yield and division into size fractions. *n* = 52–60 depending on line, tubers were harvested in total and then size fractionated

	Tuber yield (kg/plant)				
	<42 mm	42–55 mm	55–65 mm	>65 mm	Total
Kuras	0.04	0.19	0.47	0.44	1.14
8001	0.21	0.54	0.41	0.21	1.36
8003	0.35	0.40	0.11	0.01	0.87
8016	0.35	0.59	0.42	0.18	1.54
8022	0.18	0.09	0.01	0.00	0.28

For all transgenic lines, the average tuber size decreased and the fraction of tubers smaller than 55 mm increased from 35 to approximately 60% by weight of the harvested tubers for line 8001 and 8016 and more than 90% by weight for line 8003 (Table 4).

Harvested tubers from the transgenic lines showed similar phenotypic alterations as for greenhouse grown tubers with elongated shape and deeper eyes, but the characters were more pronounced (Figure S5).

**Figure 6:**
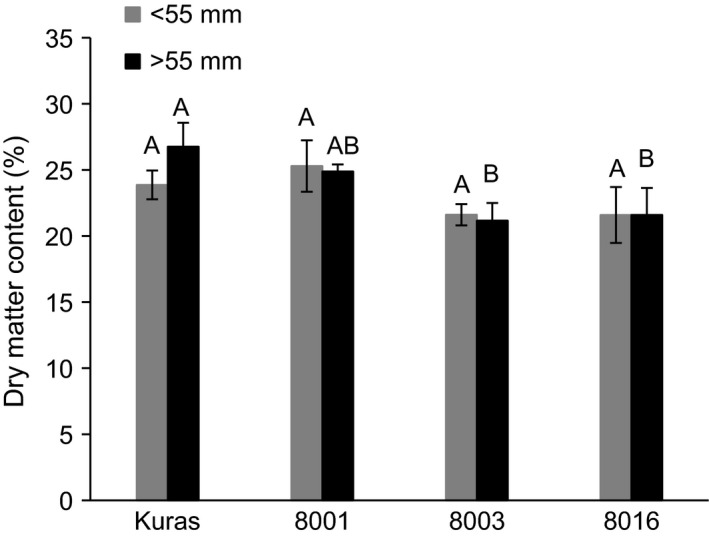
Dry matter content of field grown tubers size fractionated into <55 mm and >55 mm. Three biological replicates ±SD. Letters distinguish significant different means according to Tukey's test at level *P* < 0.05.

Dry matter content of smaller and larger fraction of tubers from transgenic lines was similar or slightly lower compared to that of Kuras (Figure 6).

### TAG and polar lipid levels and compositions are affected in field grown tubers

**Figure 7:**
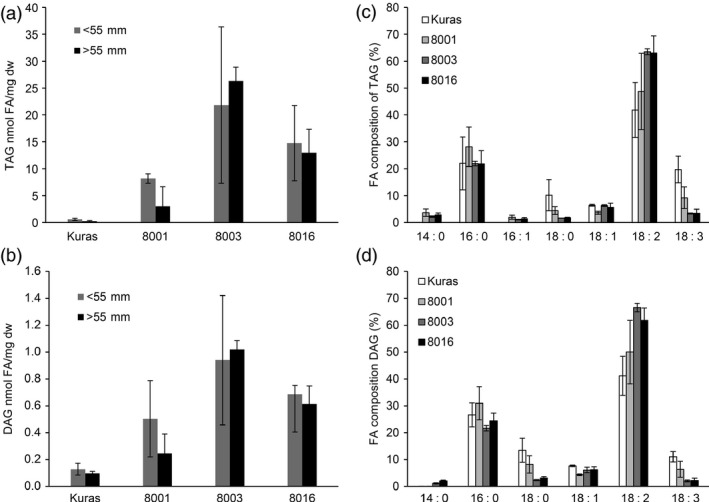
Analysis of TAG and DAG from field grown tubers, (a) TAG content of tuber of Kuras and transgenic lines, (b) DAG content of tubers of Kuras and transgenic lines, (c) TAG composition of Kuras and transgenic lines, (d) DAG composition of Kuras and transgenic lines. Three biological replicates ±SD.

TAG was found to accumulate in both smaller and larger tubers of field grown plants. Line 8003 had the highest TAG content with a 38‐fold increase in fraction of smaller tubers and a 125‐fold (0.8% of dw) increase in fraction with larger tubers (Figure 7). Diacylglycerol (DAG) levels were also higher in tubers of line 8003 with an eightfold and 10‐fold increase in respective tuber fractions over control tubers (Figure 7).

TAG composition was altered in the transgenic lines, and the most prominent change was related to higher TAG content. Fraction of 18:2 was increased from approximately 40% to around 64% of total FA in TAG, while the fraction of 18:3 was decreased. A very similar pattern could be observed for DAG composition with increases for 18:2 and decreases for 18:3 FA.

**Figure 8:**
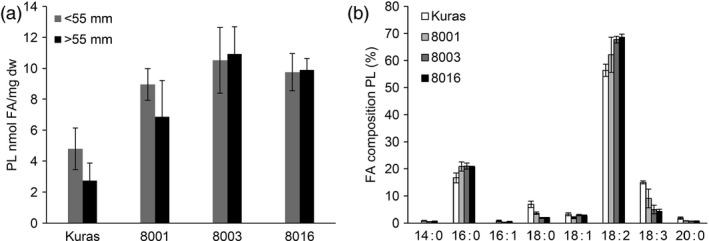
Analysis of polar lipids from field grown tubers, (a) polar lipid content of tubers of Kuras and transgenic lines, (b) Fatty acid composition of polar lipids from Kuras and transgenic lines. Three biological replicates ±SD.

Polar lipids were found to be increased in transgenic lines, and their fatty acid composition showed the same trend in the shift as for TAG although not as dramatically (Figure 8).

### Increase in tuber lipids is associated with altered starch content and composition in field grown tubers

**Figure 9:**
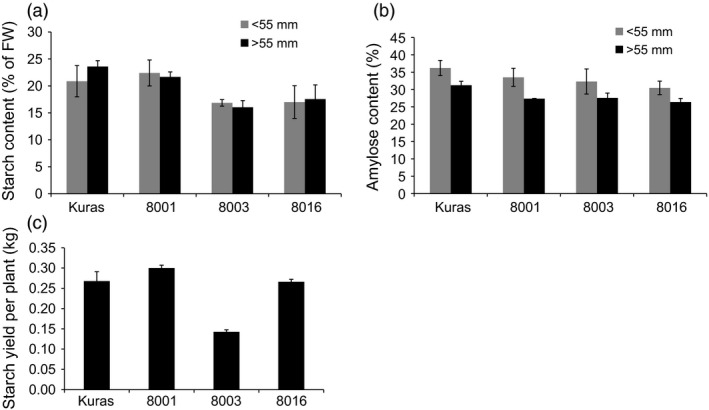
Starch content, amylose content and starch yield of field grown transgenic lines; 8001, 8003 and 8016 and parental variety Kuras. (a) Starch content (% of fresh weight), (b) Amylose ratio (% of defatted starch), (c) Average starch yield per plant (kg per plant). a‐c *n* = 3 ± SD.

**Figure 10:**
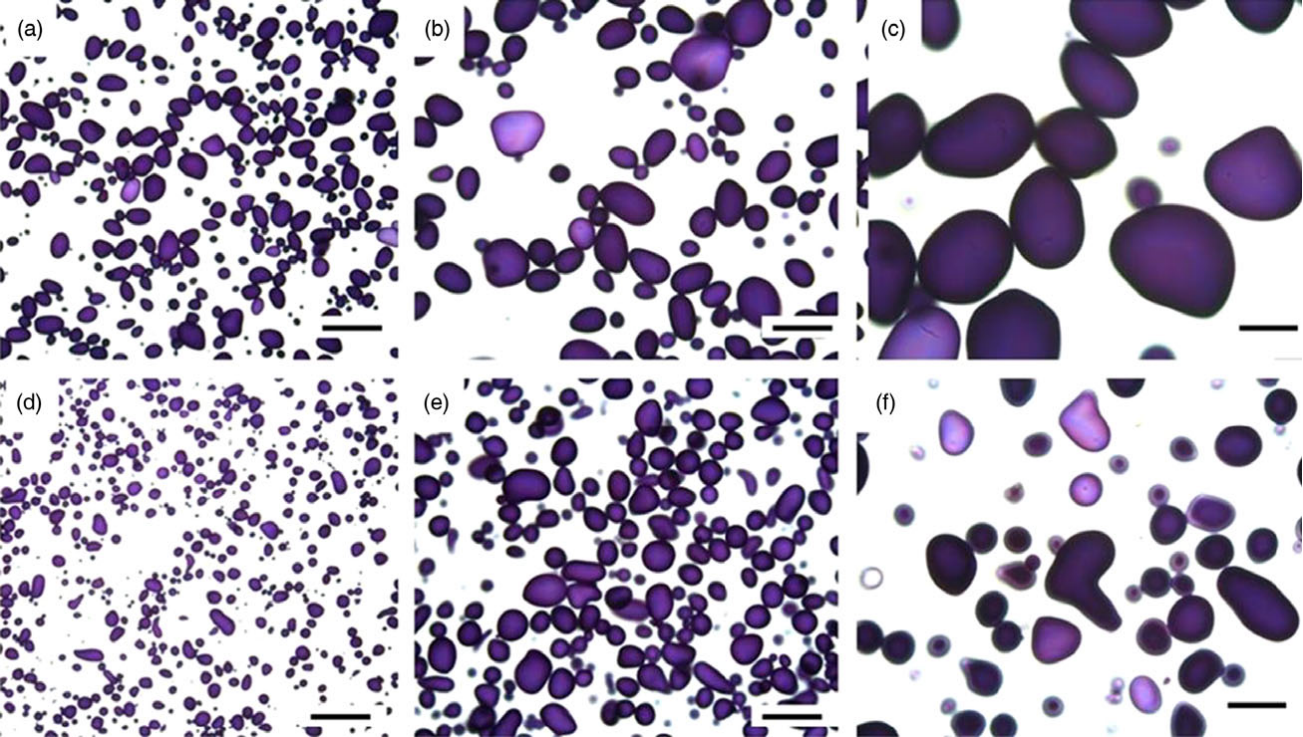
Light micrographs of starch granules of parental variety Kuras stained with iodine (a‐c) and transgenic line 8016 (d‐f). Scale bars 200 μm (a,d), 100 μm (b,e) and 50 μm (c,f).

Starch content and starch structure in tubers of the transgenic lines showed alterations in both accumulation and composition. Increased levels of tuber lipids led to a progressive decrease in starch content in almost all tuber fractions. The exception was line 8001, which showed an unaffected starch content. Starch content in both 8003 and 8016 small tuber fractions was 81% compared to wild type and only 68 and 74% compared to parental genotype in the large tuber fraction, respectively (Figure 9). The higher tuber yield in both 8001 and 8016 compensated for the low starch content, and the total starch yield per plant ended up at the same magnitude as the parental genotype (Figure 9). All transgenic lines had a higher ratio of amylopectin in the starch; however, the increase was not directly correlated to the oil content of the tubers (Figure 9). Compared to Kuras, the starch granules of 8016 were smaller and more irregular in shape (Figure 10).

### Starch‐bound neutral lipids increased in the transgenic lines

Potato starch has a very low amount of lipids bound to the starch. In the transgenic lines, the total content of neutral lipids bound to starch was drastically higher, ranging between 2 and 24‐fold increase compared to parental genotype starch (Table 5). However, the lipid content varied a lot between different tubers from the same line. The average starch‐bound lipid content of lines 8003 and 8016 was in the same range as commercially available maize starch, for which levels of 0.4% have been published (Vasanthan and Hoover, 1992).

**Table 5:** Starch‐bound lipids in field grown tubers. *N* = 3

	Starch‐bound lipids (% per mg starch)	
	Tubers < 55 mm	Tubers > 55 mm
Kuras	0.018 ± 0.007	0.017 ± 0.004
8001	0.100 ± 0.030	0.038 ± 0.019
8003	0.436 ± 0.436	0.344 ± 0.076
8016	0.339 ± 0.339	0.281 ± 0.083

## Discussion

Root and tuber crops often have a high yield per area of land and are of great importance both as food and feed and as sources for industrial raw materials. It could be attractive to supplement carbohydrate‐rich diets with fats in the harvested root or tuber crop. In addition, it could be of interest to combine starch and oil extraction which for potatoes involves a centrifugation phase where oil would go to the top and could thus easily be retrieved.

The question we wanted to answer was whether oil accumulation could be induced in potato tubers and what impact this would have on general tuber metabolism. It could be that yellow nutsedge has unique features enabling a high oil deposition in tubers, although there seem to be a big variation among different genotypes in this accumulation capacity (Matthiesen and Stoller, 1978). In a previous attempt to increase oil accumulation in potato, a single metabolic gene, acetyl‐CoA carboxylase from *Arabidopsis thaliana*, was expressed in amyloplasts of potato tubers with a fivefold increase in accumulated TAG as a result (Klaus *et al*., 2004). In the present study, a transcription factor was expressed with a potential of influencing a wider range of genes that could positively influence glycolysis and fatty acid synthesis.

A potato *GBSS* promoter was used in our study to ensure expression in tubers and a low expression in other tissues such as leaves (Visser *et al*., 1991). WRINKLED1 has been shown to negatively influence plant development if expressed in photosynthetic tissues (Cernac and Benning, 2004).

In greenhouse, transgenic lines had a higher number of tubers that in general were of smaller size with wider, deeper eye developments and frequent budding of additional tubers on the primary one. It is not directly evident what the cause for higher number of tubers and tuber size reduction might be, although it could be speculated that increased CO_2_ production or the additional metabolic and increased need of oxygen supply was causing the effect with reduced volume but maintained total tuber mass.

**Figure 11:**
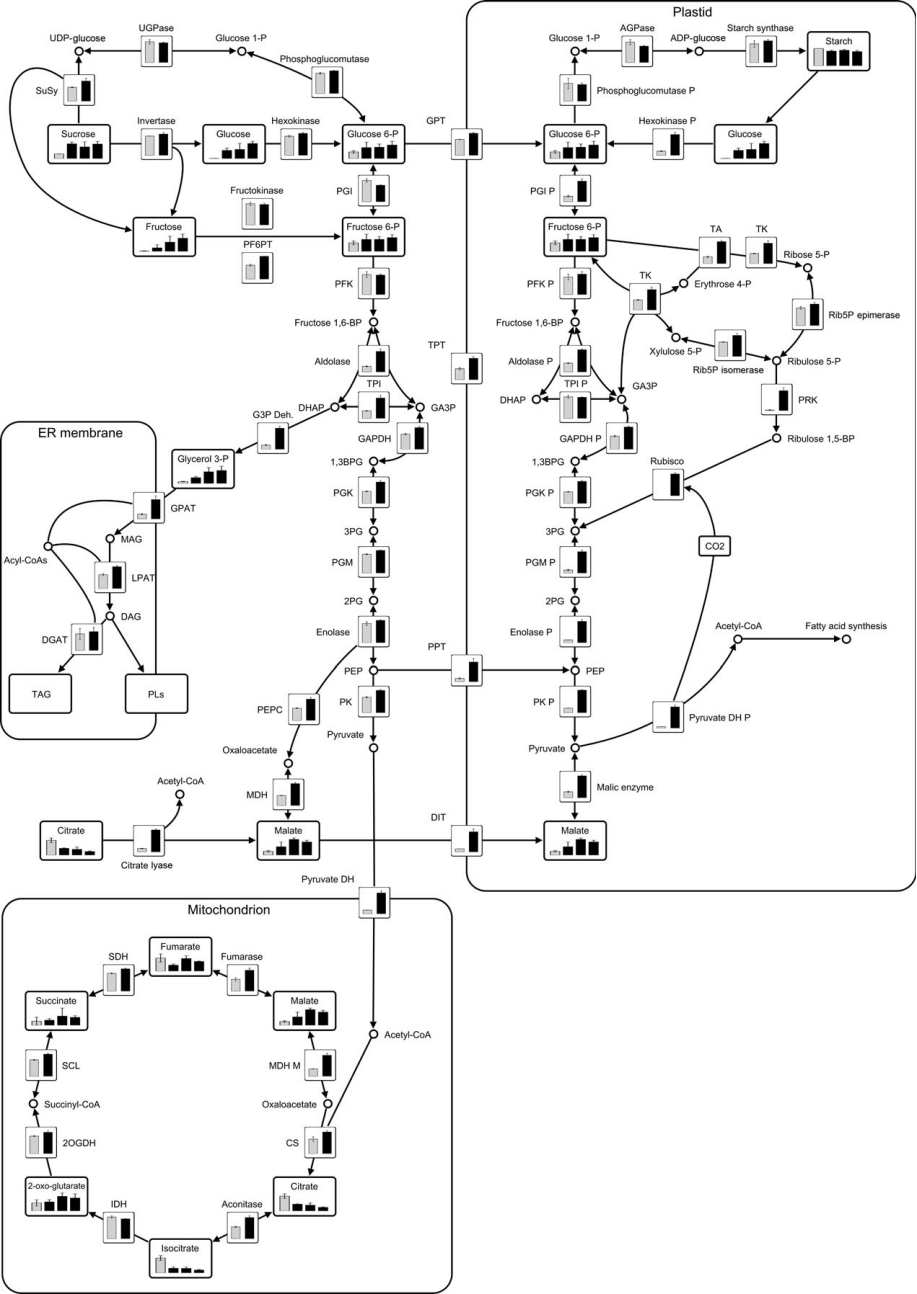
Metabolic map of greenhouse grown potato tubers: Quantified metabolites are displayed in rectangular boxes. Metabolites were analysed by GC‐MS. Kuras control tubers (grey bar) and transgenic lines 8001, 8003 and 8016 (black bars) were sampled at stage S3. Values are means of three biological replicates analysed each in two technical replicates. Error bars, standard deviations of the technical means of the three biological replicates. Transcripts are displayed in square boxes. Transcript levels of Kuras control tubers (white bar) and the transgenic line 8016 (black bar) sampled at stage S3. Transcript levels were calculated as fragments per kilobase of exon per million fragments mapped (FPKM). Values are means of three biological replicates. Error bars, standard deviation (For individual transcript levels used see Table S3). Abbreviations Enzymes: P, plastidial; M, mitochondrial; 2OGDH, 2‐oxoglutarate dehydrogenase; ACAT, Acetyl‐CoA C‐acetyltransferase; AGPase, ADP‐glucose pyrophosphorylase; CS, citrate synthase; DGAT, diacylglycerol o‐acyltransferase; DIT, dicarboxylate transporter; G3P Deh., glycerol‐3‐phosphate dehydrogenase; GAPDH, glyceraldehyde 3‐phosphate dehydrogenase; GPAT, glycerol‐3‐phosphate 2‐o‐acyltransferase; GPT, glucose‐6‐phosphate phosphate translocator; HMGR, 3‐Hydroxy‐3‐methylglutaryl‐CoA reductase; HMGS, hydroxymethylglutaryl‐CoA synthase; IDH, isocitrate dehydrogenase; LPAT, 1‐Acyl‐sn‐glycerol‐3‐phosphate acyltransferase; malic enzyme, NADP‐dependent malic enzyme; MDH, malate dehydrogenase; PEPC, phosphoenolpyruvate carboxylase; PF6PT, pyrophosphate‐fructose‐6‐P phosphotransferase; PFK, phosphofructokinase; PGI, phosphoglucose isomerase; PGK, 3‐Phosphoglycerate kinase; PGM, phosphoglycerate mutase; PK, pyruvate kinase; PPT, phosphoenolpyruvate/phosphate translocator; PRK, phosphoribulokinase; pyruvate DH, pyruvate dehydrogenase; Rib5P epimerase, ribulose‐5‐phosphate‐3‐epimerase; Rib5P Isomerase, ribose 5‐phosphate isomerase; SCL, succinyl‐CoA ligase; SDH, succinate dehydrogenase; SuSy, sucrose synthase; TA, transaldolase; TK, transketolase; TPI, triosephosphate isomerase; TPT, triosephosphate/phosphate translocator; UGPase, UDP‐glucose pyrophosphorylase. Abbreviations Metabolites: 1,3BPG, 1,3‐bisphosphoglycerate; 2PG, 2‐phosphoglycerate; 3PG, 3‐phosphoglycerate; DAG, diacylglycerol; DHAP, dihydroxacetone phosphate; GA3P, glyceraldehyde 3‐phosphate; MAG, monoacylglycerol; PEP, phosphoenolpyruvate; PL, phosphoglycolipids; TAG, triacylglycerol. Graph was created with VANTED V2.1 (Rohn *et al*., 2012).

Greenhouse grown tubers from the S3 sampling were more closely investigated as this was tubers under development and would say more about the interaction between different metabolic pathways. The TAG composition was greatly changed in transgenic tubers with an increased fraction of 18:2 primarily at the expense of 18:3. Transcripts corresponding to stearoyl desaturase and oleoyl desaturase were up‐regulated 14‐fold and eightfold, respectively, at this stage while those related to linoleoyl desaturase were essentially unchanged and furthermore expressed at very low level (Table 3, Dataset S4). This would indicate that the linoleoyl desaturation could not keep up with the increased flow of fatty acids towards TAG resulting in an increased 18:2/18:3 ratio. A prominent feature of lipid analysis from greenhouse tubers was that polar lipids increased. Polar lipids in potato tubers are mainly part of membranes and the major classes being phosphatidylcholine (PC), phosphatidylethanolamine (PE), phosphatidylinositol (PI), monogalactosyldiacylglycerol (MGDG) and digalactosyldiacylglycerol (DGDG) (Galliard, 1968). TAG is most likely produced in all tissues but generally on a very low level where most of the fatty acid synthesis in nonlipid storing tissue is sequestered to other products such as membrane lipids. *WRINKLED1* expression has been associated with large increases in TAG deposition (Baud *et al*., 2009; Bourgis *et al*., 2011; Cernac and Benning, 2004; Tranbarger *et al*., 2011). Under normal conditions, in a tissue not storing TAG, fatty acids from TAG are exposed to acyl editing and exchange with membrane lipid fractions while in oil accumulating tissue, it is to a large extent shielded from this cycling via oil body proteins organizing TAG into lipid droplets. In oil accumulating tissues, polar and membrane lipid content is rapidly passed by TAG content. The polar and membrane fatty acids found in Kuras tubers were 24‐fold in excess of TAG fatty acids but only twofold in excess in tubers of transformed lines. This indicates that although fatty acids in TAG were increased 30‐fold in transgenic potato tubers, a still much larger proportion of fatty acids stayed in polar lipids and it could be speculated that either the flux into TAG was not strong enough or budding of TAG in proper lipid bodies was not sufficiently developed which could lead to a large fatty acid exchange with polar lipids. Supply of DAG for MGDG and DGDG synthesis seemed to follow the eukaryotic pathway in potato. This means that the regulation between TAG, DAG, PC, MGDG and DGDG takes place outside the plastid where all interacting lipid species, at least theoretically, have an equal priority to increased fatty acid synthesis. It can be argued that this is a reason why the TAG must be shielded from interacting enzymes and potential shuttling between TAG and membrane lipids in nonaccumulating tissue. From transcriptomic analysis, it could be seen that genes homologous to oleosins actually did increase substantially in expression, although still to a rather low level (Table 3). Interestingly, expression of oleosins has previously not been considered as an effect of WRINKLED1, but rather transcription factors upstream as LEC2 (Santos Mendoza *et al*., 2005). From other aspects, increases of transcript types that have previously been associated with WRINKLED1 were observed, which in particular applies to genes encoding enzymes involved in late glycolysis steps and fatty acid synthesis (Baud and Graham, 2006; Cernac and Benning, 2004; Ruuska *et al*., 2002), and found much more highly expressed in the transgenic tubers (Tables 2 and 3). In particular, it was evident that transcripts associated with steps in glycolysis beyond those needed for starch synthesis were found up‐regulated. This is important as potato tubers also are sink tissues just like seeds under development, where WRINKLED1 normally is operating and there are then enzymatic steps of common importance independent of which storage compound that is accumulated. Among up‐regulated transcripts were also genes indicating metabolic activity not normally associated with WRINKLED1 but possibly a secondary effect leading towards a higher pyruvate supply. Examples are transcripts encoding enzymes in the pentose phosphate pathway (PPP) and phosphoribulokinase (PRK), ribulose‐1,5‐bisphosphate carboxylase/oxygenase (Rubisco), which together could pull metabolites from plastid hexose phosphates associated with starch synthesis (Figure 11 and Dataset S2). This observation indicated development of a Rubisco shunt which has previously been investigated in relation to CO_2_ capturing and metabolite supply for fatty acid synthesis in oil accumulating tissues (Schwender *et al*., 2004). Another observation which could indicate a change in metabolism to feed an increased need for substrates for fatty acid synthesis was the decrease in TCA cycle intermediates citrate and isocitrate concomitant with an increase in malate and expression of genes encoding ATP‐citrate lyase and malic enzyme (Figure 11). In summary, the transcriptional analysis of potato tubers expressing *WRINKLED1* displayed features going beyond what is normally associated with WRINKLED1 induction. These are likely not direct targets of WRINKLED1 but most probably secondary effects. Interestingly, a potato homolog to one of the known master regulators LEC1 was found up‐regulated in the investigated transgenic line. LEC1 is known to among others, induce oleosin encoding genes (Lotan *et al*., 1998; Mu *et al*., 2008). These, for oil accumulation, important transcripts have not been found up‐regulated upon ectopic WRINKLED1 expression even when a great number of *WRINKLED1* homologs were assessed in leaf tissue (Grimberg *et al*., 2015).

Surprisingly concomitant with an increase in TAG accumulation, an almost 100‐fold increase of glucose to more than 18% of dry weight was found. This was accompanied by a large increase of also sucrose and fructose but to more moderate absolute levels and could indicate an increased mobilization of starch. However, in the transcriptional changes caused by *WRINKLED1* expression, there were no obvious clues which could explain this increase in sugars although many enzymes participating in carbohydrate and starch metabolism are subject to post‐translational modifications as phosphorylation and redox modifications, which regulate the activity of and interaction between enzymes (Geigenberger, 2011).

The potato lines were subjected to a field trial where tubers were planted and harvested following a general time schedule for starch potatoes. Tubers were divided into two fractions of being either <55 mm in size or larger than 55 mm in size. Trends were similar to that of greenhouse tubers in that total tuber yield increased and tuber sizes decreased in comparison with control. Tuber phenotype was altered as for greenhouse tubers but with a tendency for more elongated tubers with wider and deeper eyes. Alterations in tuber phenotype have previously been observed in connection with modification of the starch metabolism resulting in a decrease in starch content and an increase in soluble sugars (Hofvander *et al*., 2004; Riewe *et al*., 2008; Tjaden *et al*., 1998). The tubers in these published studies did show an elongated shape and a tendency to bud‐off additional tubers.

Individual samples of field grown tubers were found to have TAG content of up to 1.1% of dry weight, although the averages of the field grown biological replicates were in a similar range to those found in greenhouse tubers under development. Thus at least seemingly, TAG content of field grown tubers was maintained contrasting the situation in greenhouse tubers where the concentration decreased between the S3 and S4 sampling.

That starch accumulation and organization were affected by the novel metabolic routes could be seen from both content and also from that the starch composition was slightly altered with lower amylose content and that the shape and size of starch granules were impacted resulting in smaller granules and occasional prominent extrusions extending out from the granules. An open question is if the observed increased level of starch‐bound lipids could cause any of these effects.

This study showed that accumulation of triacylglycerols with an altered composition in potato tubers could be induced by the *Arabidopsis thaliana* transcription factor WRINKLED1. It was clear from this study that a starch storing tuber crop as potato does not have a dormant capacity for oil accumulation in the form of TAG at least not by up‐regulating basic factors for fatty acid synthesis. One factor of importance that could be identified was that unlike in storage organs which primarily store TAG, there was a concomitant increase of membrane lipids. This deposition of additional fatty acids could also disturb tuber development. Commonly, TAG produced in oil‐storing tissue is organized by oil body proteins which lead to structures of a defined size that also are shielded and protected from other metabolic activities. From structural studies of the transgenic tubers, there was an indication of oil in droplets which would lead to the conclusion that proteins for organization into oil bodies are present in the tubers. This is similar to the generation of starch granules during starch accumulation which also is a way to pack the deposition product and shield it from other metabolic activities. Thus, as further studies, it would be of great interest to overexpress genetic factors which could protect the accumulated TAG and reduce interactions with membrane lipids via PC or increase the pull towards TAG via diacylglycerol acyltransferase (DGAT) as has been carried out in transgenic tobacco (Vanhercke *et al*., 2014). To fully utilize the storage product accumulation capacity of potato and potato tubers, another crucial topic for further studies is which mechanism or mechanisms that trigged or caused the huge accumulation of sugars as the concentration increase in itself is very likely to interfere with efficient sink mechanisms and storage product accumulation.

## Experimental procedures

### Gene construct

A synthetic gene corresponding to *AtWRINKLED1* (Cernac and Benning, 2004) was ordered (Eurofins/MWG, Ebersberg, Germany) and cloned after a 990 bp *Solanum tuberosum GBSS* promoter fragment (Hofvander *et al*., 2004) in a pBIN19 (Bevan, 1984) derivative binary vector. After controlling the integrity of the plasmid, it was transformed into *Agrobacterium tumefaciens*, strain *AGL0* (Lazo *et al*., 1991) via direct transformation (An *et al*., 1989).

### Plant material and growth conditions

Leaf tissue of potato cultivar Kuras was transformed as previously described (Andersson *et al*., 2003), but with 50 mg/L kanamycin as selection agent. Regenerated shoots were analysed for the presence of *nptII* and the absence of *Agrobacterium virG* sequence using REDExtract‐N‐Amp Plant PCR Kit (Sigma‐Aldrich, St Louis, MO). Primers; nptIIf 5'‐CCTGTCATCTCACCTTGCTC‐3′, nptIIr 5'‐AGTCCCGCTCAGAAGAACTC‐3', VirG‐ 5′‐GCCGGGGCGAGACCATAGG‐3′ and VirG+ 5′‐ CGCACGCGCAAGGCAACC‐3′(Sigma‐Aldrich).

Cuttings were propagated to produce microtubers (4.4 g/L MS medium, 2.5 mg/L kinetin, 0.5 mg/L abscisic acid (ABA), 8% sucrose and 200 mg/L cefotaxime at 22°C) in dark.

Greenhouse trial was performed from mid of August to mid of December, with 5–10 pots per line, in 7.5 L pots at 16 h day length, 18/15°C day/night temperature, supplementary light intensity of approximately 200 μmol/s/m^2^ photons and 60% relative humidity. Generally, three tubers were pooled to yield one biological replicate. Tubers were stored at 8°C for 4 months and used as seed potato for field trial. About 52 or 60 seed tubers per line were planted and grown in field close to Kristianstad (Skåne, Sweden) between 14th of May and 17th of October. The plants were grown in plots of 15 or 13 plants per row in four rows. The distance between the plants was 32 cm, and the distance between the rows was 75 cm. Line plots harvested after senescence as one sample and size fractionated. Generally, three tubers were pooled to yield one biological replicate.

### Determination of copy number by Southern blotting

Genomic DNA was isolated using Illustra DNA Extraction kit Phytopure (GE Healthcare, Buckinghamshire, UK). About 10 μg DNA was digested with EcoRV and separated on a 1% agarose gel and blotted to a Hybond N+ membrane (GE Healthcare). A 498 bp npt II PCR product amplified with primers 5′‐ CCTGTCATCTCACCTTGCTC ‐3′ and 5′‐AGTCCCGCTCAGAAGAACTC ‐3′ and labelled with PCR DIG Probe synthesis kit (Roche Diagnostics GmbH, Mannheim, Germany) was used as probe and hybridized to the membrane o/n. CDP‐*Star,* ready‐to‐use (Roche Diagnostics GmbH, Mannheim, Germany) was used for detection of the hybridized probe and exposed to an X‐ray film.

### Lipid analyses

Tuber samples were freeze‐dried (Coolsafe, Scanvac, Lynge, Denmark). Total lipids were extracted (Bligh and Dyer, 1959), and the CHCl_3_ extracts were subsequently separated (aliquots corresponding to 100 mg fw) using thin layer chromatography on TLC Silica gel 60 (Merck, Darmstadt, Germany). For neutral lipids, 70:30:1 heptane:diethylether:HAc was used as a mobile phase and for polar lipids 95:15:10:3 CHCl_3_:MeOH:HAc:H_2_O. Fatty acid methyl esters were quantified using gas chromatography as previously described (Leonova *et al*., 2008).

### Quantitative and qualitative starch determination

Dry matter was determined after 3 days of freeze drying (Coolsafe, Scanvac, Lynge, Denmark). Homogenized freeze‐dried tuber tissue was used for measurement of total starch content using a Total starch kit (Megazyme, Bray, Co. Wicklow, Ireland). Samples were washed with 80% ethanol to remove glucose present in the sample. For calculation of starch content, a standard curve was used based on a maize starch sample supplied in the kit.

Starch was isolated and purified from fresh tubers (Larsson *et al*., 1996). Amylose content of isolated starch was measured according to a DMSO and iodine‐based colorimetric analysis method (Andersson *et al*., 2006).

Starch‐bound lipids were extracted from purified starch using a water‐saturated n‐propanol extraction method (Vasanthan and Hoover, 1992).

### Extraction, determination and quantification of metabolites

For the metabolite measurements by GC‐MS analysis, 20 mg of flash‐frozen and lyophilized potato tuber material was ground to a fine powder using a shaking mill and glass beads (5 mm). The polar fraction was extracted and derivatized with 30 μL methoxyamine hydrochloride and 60 μL N‐methyl‐N‐(trimethylsilyl) trifluoroacetamide (MSTFA) as previously described (Bellaire *et al*., 2014) to transform the metabolites into their methoxyimino (MEOX)‐ and trimethylsilyl (TMS)‐ derivatives. *allo*‐Inositol was used as an internal standard. The samples were analysed on an Agilent 5973 Network mass selective detector connected to a Agilent 6890 gas chromatograph equipped with a capillary HP5‐MS column (30 m × 0.25 mm; 0.25 μm coating thickness; J&W Scientific, Agilent, Santa Clara, CA). Helium was used as carrier gas (1 mL/min). The inlet temperature was set to 230°C, and the temperature gradient applied was 50°C for 2 min, 50–330°C at 5 K/min 330°C for 2 min. Electron energy of 70 eV, an ion source temperature of 230°C and a transfer line temperature of 330°C were used. Spectra were recorded in the range of 71–600 da/e.

Masses used for quantification of the individual metabolites are depicted in Dataset S1. For absolute quantification, all compounds were quantified against the standard using a calibration curve performed with pure substances based on a total of 12 measurements for each substance.

### Structural analysis of tuber tissue

For fixation and plastic embedding, tubers with an approximate weight of 10 g were harvested from pots grown in greenhouse. Tissue samples were taken from six different locations in the tuber, and orientation of the samples in relation to the stolon was noted. The tissue was immediately aldehyde‐fixed, post‐fixed in osmium tetroxide and embedded in fresh Spurr's resin (Low viscosity kit; Ted Pella, Redding, CA) (Turesson *et al*., 2010).

For transmission electron microscopy, ultrathin sections were cut from Spurr‐embedded blocks and handled as in Turesson *et al*. (2010). A JEM‐1010 electron microscope (JEOL, Tokyo, Japan) with an accelerating voltage of 60 kV was used to examine the sections.

For staining of light micrographs, an overview was obtained by staining 1‐μm‐thin sections of Spurr‐embedded tuber tissue with MAS (Triple staining methylene blue‐azur A‐safranin O), visualising proteins, lipids and starch (Heneen *et al*., 2008; Turesson *et al*., 2010; Warmke and Lee, 1976). Sudan Black was used for staining oil and lipids as described earlier (Heneen *et al*., 2008; O'Brien and McCully, 1981; Turesson *et al*., 2010). The stained and air‐dried sections were mounted with Biomount (British Biocell, Cardiff, UK) and studied in a light microscope (Leica Microsystems, Wetzlar, Germany).

For starch staining of homogenized tissue, Lugol's solution (Scharlau, Sentmenat, Spain) and glycerol (1:1) was added to purified starch granules and studied under a light microscope (Leica Microsystems, Wetzlar, Germany).

### Transcriptome analysis

Tuber tissue from line 8016 and Kuras was sampled from greenhouse grown plants at three different time points, 2, 3 and 4 months after planting (S2, S3 and S4, respectively), and immediately flash‐frozen in liquid nitrogen. Tissue from three tubers grown in individual pots was pooled prior to total RNA extraction using Plant RNA Reagent (Invitrogen, Life technologies Ltd, Waltham, MA) according to the manufacturer's instructions. This was applied in triplicates as biological replicates for all three stages. Quality and quantity of the RNA samples were measured on a NanoDrop (NanoDrop^™^ 1000 Spectrophotometer, Thermo Scientific, Waltham, MA) and using electrophoresis on a 1.2% E‐gel (Invitrogen, Life Technologies Ltd).

Library construction and sequencing using Illumina HiSeq technology was performed by GATC Biotech (Constance, Germany).

All the prepared samples were sequenced through Illumina sequencing platform HiSeq 2000 as unpaired‐end reads with 50‐bp read length. Reads were trimmed by removing adapter sequences, and low quality sequences were removed. Assembly was carried out with high‐quality reads using Trinity version trinityrnaseq_r2014‐07‐17 with default parameters (Haas *et al*., 2013).

To assess the quality of the transcriptome assembly, reads were mapped back to the assembled transcripts using the Bowtie2 aligner (Langmead and Salzberg, 2012). The mapping results were visualized using Integrated Genomics Viewer version 2.3.2 (IGV) (Thorvaldsdottir *et al*., 2013). The completeness of the transcriptome assembly was estimated with CEGMA version 2.4.010312 software (Parra *et al*., 2007). CEGMA analysis was performed on default parameters.

High‐quality‐assembled transcriptome was annotated with BLAST2GO (Conesa *et al*., 2005). BLASTX was used against the NCBI nonredundant (nr) protein database using an e‐value cut‐off of 1E‐5. InterProScan was used to identify the conserved domains/motif for each transcript.

RSEM version 1.2.15 was used for abundance estimation of the assembled transcripts using the default parameters (Li and Dewey, 2011). The relative measure of transcript abundance was TPM (transcripts per million) and FPKM (fragments per kilobase of transcript per million mapped reads). The relative level of all assembled transcripts can be found in Dataset S4. EdgeR Bioconductor (Robinson *et al*., 2010) was used in identification and analysis of differentially expressed genes and transcripts through Trinity versiontrinityrnaseq_r20140717 on default settings. Data on all transcripts determined as differentially expressed can be found in Dataset S3.

## Conflict of interest

All authors declare no conflict of interest.

## Supporting information


**Figure S1** TLC separation of neutral lipids extracted from microtubers of Kuras, transgenic lines and potato seed.
**Figure S2** Triacylglycerol content of microtubers representing Kuras and transgenic lines 8001 and 8016.
**Figure S3** Fatty acid composition of triacylglycerol from Kuras control microtubers, microtubers of transgenic lines 8001 and 8016, potato seed.
**Figure S4** Southern blotting of total DNA from Kuras and transgenic potato lines.
**Figure S5** Field grown tubers of transgenic line 8016.

**Dataset S1** Metabolites shown in Figure 11.

**Dataset S2** Transcripts shown in Figure 11.

**Dataset S3** Transcripts differentially expressed in transgenic line 8016.

**Dataset S4** Expression levels of transcripts in Kuras and transgenic line 8016.
