# Supplementary material for: Potato tuber expression of Arabidopsis WRINKLED1 increase triacylglycerol and membrane lipids while affecting central carbohydrate metabolism
Source: Plant Biotechnol J. 2016 Mar 17;14(9):1883–98. doi: 10.1111/pbi.12550 (PMC5069604; doi:10.1111/pbi.12550)
Supplement: Supplementary file 1 — Figure S1 TLC separation of neutral lipids extracted from microtubers of Kuras, transgenic lines and potato seed. Figure S2 Triacylglycerol content of microtubers representing Kuras and transgenic lines 8001 and 8016. Figure S3 Fatty acid composition of triacylglycerol from Kuras control microtubers, microtubers of transgenic lines 8001 and 8016, potato seed. Figure S4 Southern blotting of total DNA from Kuras and transgenic potato lines. Figure S5 Field grown tubers of transgenic line 8016. [file PBI-14-1883-s005.docx]

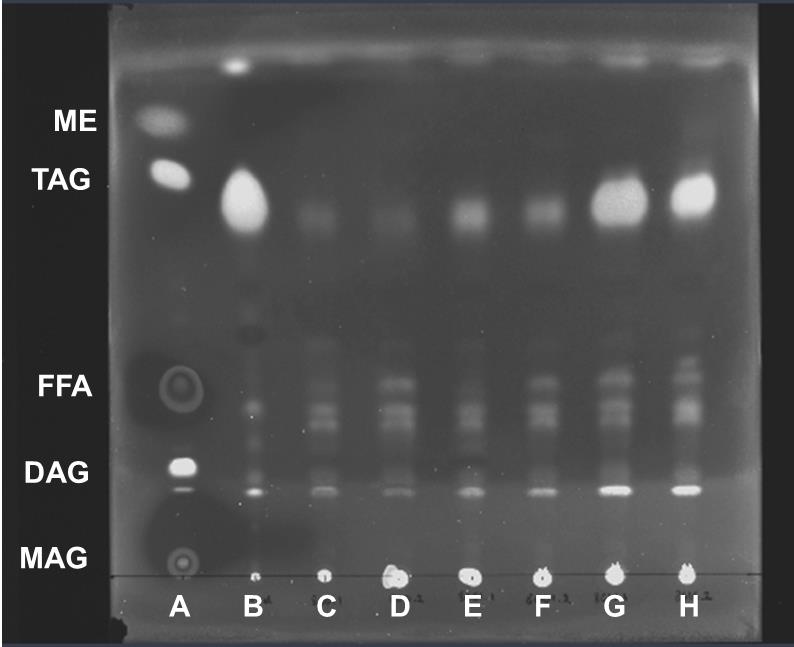


Figure S1. A. Neutral lipid standard, B. Potato seed lipid, C. and D. Kuras microtuber, E. and F. Line 8001 microtuber, G. and H. Line 8016 microtuber. Lipid extract corresponding to 0.57 mg seed and 15 mg fw microtuber was applied. Tubers were derived from individual plantlets.


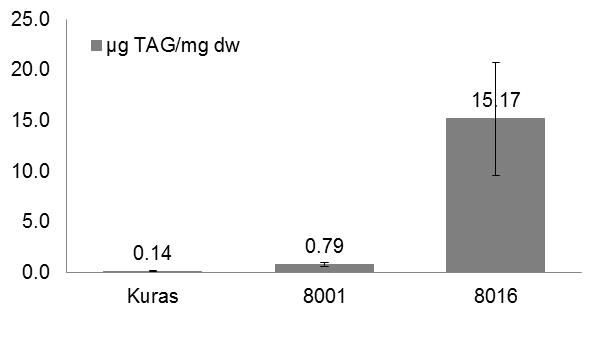


Figure S2 Triacylglycerol content of microtubers representing Kuras and transgenic lines 8001 and 8016.


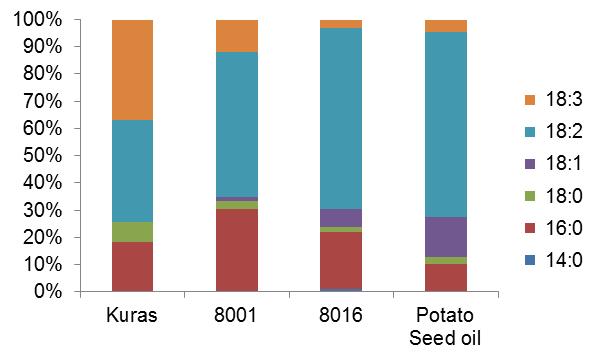


Figure S3 Fatty acid composition of triacylglycerol from Kuras control microtubers, microtubers of transgenic lines 8001 and 8016, potato seed.


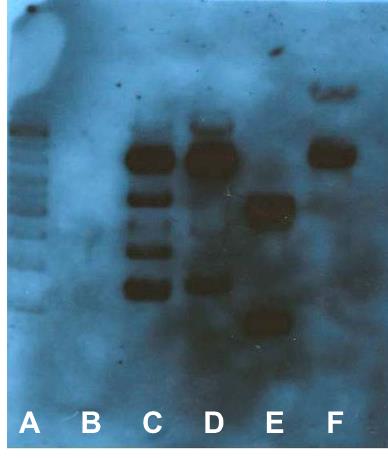


Figure S4 Southern blotting of total DNA from potato lines digested by EcoRV using an nptII fragment as a probe, A. Molecular marker, B. Kuras, C. 8001, D. 8003, E. 8016, F. 8022.


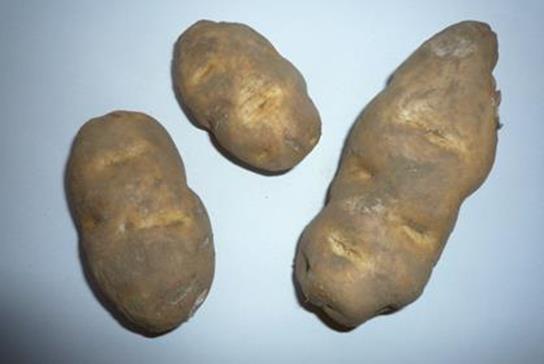


**Figure S5** Field grown tubers of transgenic line 8016. Solid arrows indicate deep eyes of transgenic tubers.
